# Examining Science Practices in an Introductory Biology Laboratory Curriculum

**DOI:** 10.1093/iob/obag049

**Published:** 2026-08-18

**Authors:** C L Dahlberg, K Hunter, L Spring, E Worline, J Weaver, K Bolland, E Duffy, T Huỳnh, N Stephenson

**Affiliations:** Biology Department, Western Washington University, Bellingham, WA 98225, USA; Department of Chemistry, Western Washington University, Bellingham, WA 98225, USA; Current Institution: University of Montana, 32 Campus Drive, Missoula, MT 59812, USA; Biology Department, Western Washington University, Bellingham, WA 98225, USA; Biology Department, Western Washington University, Bellingham, WA 98225, USA; Biology Department, Western Washington University, Bellingham, WA 98225, USA; Science, Math, and Technology Education Western Washington University, Bellingham, WA 98225, USA; Department of Chemistry, Western Washington University, Bellingham, WA 98225, USA; Science, Math, and Technology Education Western Washington University, Bellingham, WA 98225, USA; Current Institution: Solebury School, 6832 Phillips Mill Rd, New Hope, PA 18938, USA; Department of Physics & Astronomy, Western Washington University, Bellingham, WA 98225, USA; Science, Math, and Technology Education Western Washington University, Bellingham, WA 98225, USA; Department of Chemistry, Western Washington University, Bellingham, WA 98225, USA; Science, Math, and Technology Education Western Washington University, Bellingham, WA 98225, USA

## Abstract

The three-dimensional (3D) learning model for science teaching and learning introduced in *A Framework for K-12 Science Education* and the Next-Generation Science Standards (NGSS) provide guidance towards including science practices in biology coursework. Science practices incorporate both content knowledge and skills and describe activities that scientists routinely engage in. The 3D-Learning Assessment Protocol (3D-LAP) provides criteria required for each science practice. We used the 3D-LAP to evaluate the extent to which three linked introductory biology laboratory courses have the potential to engage students in science practices. In the three courses, we found that 24/25 of the lab sessions had the potential to engage students in at least one science practice, with a total of 152 instances over the three courses. There was strong emphasis on four practices: Analyzing and Interpreting Data, Developing and Using Models, and Constructing Explanations and Engaging in Argumentation from Evidence. However, three practices–Planning Investigations, Communicating Information, and Defining Problems/Designing Solutions–were not identified in any of the biology laboratory sessions. In the courses we investigated, we also found that the potential for students to engage in science practices would increase by 89% if activities that met all but one criterion from the 3D-LAP (“*near misses*”) were modified so that all criteria were met. Based on our assessment, if *near misses* were available in these courses, students would have the opportunity to engage in two science practices that are otherwise absent from the laboratory curriculum. We propose that instructors can align introductory-level, course-based laboratory work with the criteria in the 3D-LAP. A practice-focused curricula can help students gain technical facility and participate in science practices in ways that also support the *Core Competencies* described in *Vision and Change*.

## Introduction

**Fig. 1:**
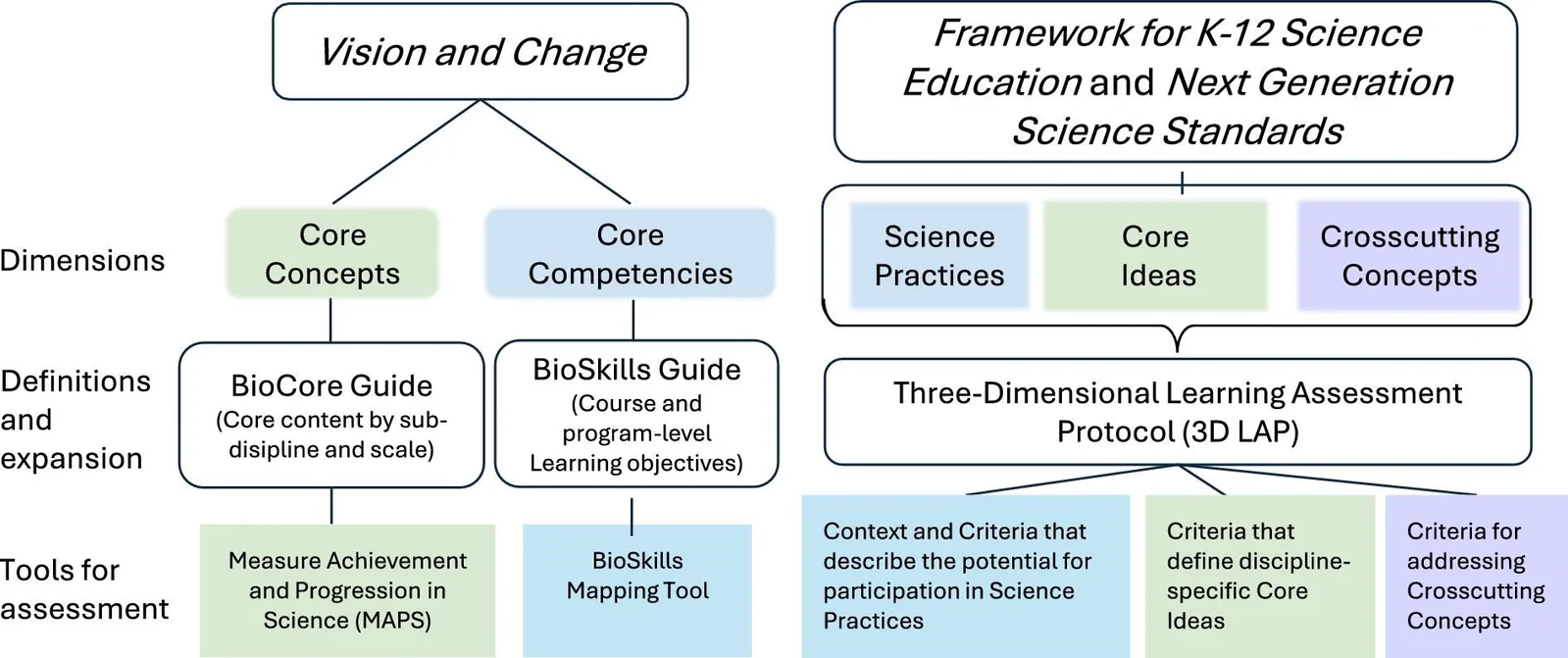
Visual guide showing the parallels between the core concepts and core competencies described by vision and change, and the science practices, core ideas, and crosscutting concepts outlined in *The Framework for K-12 Science Education and Next Generation Science Standards* (AAAS 2011, National Research Council 2012, National Research Council 2013). Color coding illustrates the parallel focus on content (light green) and practice (light blue) in the two frameworks. The categories in *Vision and Change* were created with Biological Science in mind and have been further developed and expanded by the BioCore Guide and BioSkills guide ((Brownell and Tanner 2012, Clemmons et al. 2019), 63). The three dimensions of learning described by *The Framework* and NGSS address all physics, chemistry, and biology. Tools for assessment include the sub-disciplinary MAPS and BioSkills mapping tool and the categories for assessment included in the 3D-LAP (Laverty et al. 2016, Couch et al. 2017, Summers et al. 2018, Carmel et al. 2019, Semsar et al. 2019, Clemmons et al. 2020). In this study, we have focused on Science Practices in introductory biology laboratory courses, which often align with the BioSkills Guide’s Learning objectives.

Defining how and what undergraduate students should learn in a biology curriculum has long been debated and remains an important topic for science educators. Over the past several decades, numerous reports and frameworks, each emphasizing different aspects of biology education, have proposed standards for undergraduate biology learning. Perhaps the most impactful of these documents is AAAS’s *Vision and Change* which formalized a set of knowledge and skills (Core Concepts and Core Competencies) for graduating biology undergraduates that have been adopted by many biology educators (AAAS 2011; Hsu et al. 2025). In addition to *Vision and Change*, which is biology specific, *A Framework for K-12 Science Education* (hereafter referred to as *The Framework*) and the Next Generation Science Standards (NGSS) provide guidance for instructors that encompasses science disciplines including biology (National Research Council 2012; National Research Council 2013). *The Framework* and the NGSS introduce the three-dimensional (3D) learning model which promotes the integration of disciplinary core ideas, crosscutting concepts, and science practices for deep and meaningful learning. Although the 3D learning model was originally designed with a focus on K-12 education, it is suitable for and has been used in early-career undergraduate education and beyond (Laverty et al. 2016; Underwood et al. 2016; Carmel et al. 2017; Matz et al. 2018; Carmel et al. 2019; Stephenson et al. 2020, Bain et al. 2020; Stephenson et al. 2023; Van Wyk et al. 2025). Figure 1 shows the learning goals of *Vision and Change, The Framework*, and the NGSS, and highlights the parallels between them.

Of the three dimensions in the 3D model, we are particularly interested in the science practices–processes that scientists regularly engage in as they carry out their work–and how opportunities for students to engage in these practices show up in introductory biology laboratory curricula. *The Framework* defines eight science and engineering practices–Asking Questions, Developing and Using Models, Planning and Carrying Out Investigations, Analyzing and Interpreting Data, Using Math and Computational Thinking, Constructing Explanations, Engaging in Argument from Evidence, and Obtaining, Evaluating and Communicating Information. These were later disaggregated into ten practices by Carmel et al. for assessing laboratory activities (Carmel et al. 2019). Engaging in science practices requires access and use of both content knowledge and skills simultaneously, and as they describe behaviors and activities that scientists engage in as part of their work, they are critical for involving students in “doing science” (National Research Council 2012; Laverty et al. 2016; Carmel et al. 2019).

Since the introduction of *The Framework* and NGSS, research into the science practices in the biological sciences has largely centered around how instruction can support student engagement in practices (e.g., Zagallo et al. 2016; Bierema et al. 2017; Bolger et al. 2021; Gouvea et al. 2022; Killpack and Popolizio 2023; Honig et al. 2025) and how science practices are assessed (e.g., Zagallo et al. 2016; Bennett et al. 2020; Paine and Knight 2020; Gouvea et al. 2022; Uminski et al. 2024). Many of these works have revealed that instruction that is not intentional about including science practices does not do well at providing opportunities for students to engage in practices. For example, Uminski and colleagues examined biology exam questions from 100 institutions across the United States and found that only a small percentage (~7%) of these questions could elicit a science practice, with about one-third eliciting no practice at all (Uminski et al. 2024). However, Honig and colleagues found that a curriculum that was adapted to intentionally provide students with opportunities to engage in the science practices of generating scientific explanations had more success than a traditional curriculum (Honig et al. 2025). Research in chemistry and physics laboratory courses also reveal the need for intentional curriculum design towards alignment with science practices as an important factor in ensuring that laboratory materials have the potential to engage students in science practices (e.g., Stephenson et al. 2020; Chen and Terada 2021; Hosbein and Walker 2022; Polk and Santos 2025; McFall-Boegeman et al. 2025; Bolland et al. 2025; Stephenson et al. 2026). While some of the research into science practices in the biological sciences focused on the introductory laboratory (Paine and Knight 2020) or on laboratory reports (Gouvea et al. 2022), these have primarily centered on a single practice or a select set of practices (Chi et al. 2026).

Although science practices are not confined to laboratory settings and are routinely used by scientists in and out of the lab, laboratory classrooms provide a space for “hands-on, heads-on” learning that combines technical practice with application of content knowledge (National Research Council 2012), so they are especially well-suited to engaging students in science practices. For this study, we assessed a series of three introductory biology courses. In this series, students attend laboratory sessions for approximately 40% of their scheduled instruction time, and the lab sessions have a 3–4x higher instructor to student ratio, compared to the classroom sessions. Thus, there is a significant time and resource commitment for students to engage in meaningful learning during lab participation. Given the potential for students to engage in technical practice coupled with application of content instruction, we were interested in whether students had the opportunity to engage in science practices in the laboratory sections of their coursework, as opposed to being asked to focus mainly on developing technical skills in the lab.

To explore how opportunities to engage in science practices show up in the introductory biology lab curriculum, we employed a tool called the Three-Dimensional Learning Assessment Protocol (3D-LAP) (Laverty et al. 2016). The 3D-LAP was originally developed to determine whether lecture assessment materials met the criteria for learning across the three dimensions (disciplinary core ideas, science practices, and crosscutting concepts) laid out in *The Framework* and NGSS. Although the 3D-LAP was designed for use with lecture assessments, it has also been used in laboratory coursework (Carmel et al. 2019; Stephenson et al. 2023) and has been applied for identification of science practices, separately from the other two dimensions (Carmel et al. 2019; Stephenson et al. 2023; Uminski et al. 2024; McFall-Boegeman et al. 2025; Bolland et al. 2025; Stephenson et al. 2026). In addition to complete presence or absence of science practices, previous studies have also observed challenges in meeting *all* criteria of science practices, such as a “partial alignment” with the defined criteria (Uminski and Couch 2024) or a fulfillment of “all but the last criterion” (Van Wyk et al. 2025).

In this article, we describe the results of our evaluation of the laboratory manuals and associated pre- and post-lab assignments in three introductory biology laboratory courses. We focus on the opportunities for engagement in science practices provided by these laboratory materials and report on which science practices are present and absent from this introductory curriculum, and where science practices show up (that is, in or out of lab). We also present the landscape of materials that do not fully meet the criteria required to elicit science practices, which we term “*near misses”* and describe how relatively minor changes could transform the curriculum by modifying *near misses* so that they meet all of the criteria required to elicit science practices. Our analysis highlights how introductory biology laboratory courses can encourage early-stage undergraduate students to engage in labs as *scientists*.

To our knowledge, there is no other work that has examined how the science practices are situated in a full introductory biology laboratory curriculum using the well-defined practices in *The Framework* and the 3D-LAP and its extensions. Although this work examined the lab curriculum at a single institution, it provides important information to the community about what opportunities for engagement in the full spectrum of science practices are afforded by a typical traditional curriculum, and what implications this has for achieving learning outcomes, and for teaching and learning broadly.

## Methods

This study was performed at a predominantly undergraduate institution (PUI) that serves more than 14,000 undergraduate students on the academic quarter system. Each quarter has 10 weeks of academic instruction, followed by a final exam period. We assessed the introductory biology series (BIOL 1, BIOL 2, and BIOL 3), which takes three quarters of instruction (one academic year) to fulfill. The courses cover a wide variety of topics: Evolution, Ecology, and Biodiversity (BIOL 1), Cellular and Molecular Biology (BIOL 2), and Organismal Biology (BIOL 3). The biology department offers this single curriculum for introductory study that applies towards degrees in biology but also serves students from other majors. The introductory biology courses include large lecture sections (up to 96 students) that are subdivided into smaller laboratory sections (24 students per section). While laboratory sections may be taught by different instructors, the same materials are used in all sections of the same course. Typical annual enrollment in the introductory biology courses is high: about 700 students in BIOL 1, with slightly lower annual enrollment in subsequent introductory courses (BIOL 2 and BIOL 3).

This study was carried out as part of a larger study into science practices in introductory laboratory courses across three disciplines: biology, chemistry, and physics. The research was carried out in accordance with the protocol approved by the Institutional Review Board. None of the authors were instructors for the introductory biology series during the span of the study, though the authors CLD, NS, and TH teach in the biology, chemistry, and physics departments, respectively. Student authors (coders) had all taken at least one of the BIOL courses in the series. One author (CLD) taught the BIOL 2 course and contributed some revisions to the manuals ten years prior to the beginning of this research project. This study does not report on any student work or observations of students in the laboratory.

We used an extended version of the 3D-Learning Assessment Protocol (3D-LAP) (Carmel et al. 2019) to examine laboratory materials (manuals, pre-labs and post-labs) and identify the science practices that students were asked to engage in during the laboratory sessions. The 3D-LAP defines 2–4 criteria to characterize the potential of college science assessments to elicit student engagement in disciplinary core ideas, crosscutting concepts and science (and engineering) practices (Laverty et al. 2016). The 3D-LAP was originally used to characterize non-laboratory college science assessments for potential to engage students in disciplinary core ideas, cross cutting concepts and science practices. Carmel and colleagues modified the 3D-LAP criteria for science practices for use in laboratory settings and we used this extended version of the 3D-LAP for for science practices to assess introductory biology laboratory materials^1^ (Carmel et al. 2019).

Table 1 provides an overview of the three-hour laboratory sessions (one session per week for nine weeks) along with the practices that were identified for that session. The lab sessions are complementary to the lecture portion of the courses. Generally, each course includes an early introduction to various scientific techniques and instrumentation that are used throughout the course or courses (compound and dissection microscopy, computer modeling and bioinformatic tools, spectrophotometry, serial dilution, dissection). Each lab session has one or two major foci (Laboratory content) that students interact with (Student Actions). The coursework in these labs does not include any Course-based Undergraduate Research Experience (CURE) curriculum.

**Table 1:** Overview of laboratory activities in an introductory biology curriculum

Lab #	Title	Laboratory content		Student actions	Practices coded
**BIOL 1**					
Lab 1	Process of Science	Gaining technical proficiency with a compound microscope	Setting up a long-term experiment on the interaction between mycrorhyzae and plant growth	Students gain familiarity with scientific instrumentation and experimental organisms (seedlings).	P1, P4
Lab 2	Mitosis, Meiosis, and Mendel	Modeling the cell cycle, ploidy and cell division	Describing [independent] chromosomal segregation	Students use Punnett squares and whiteboards to explore chromosomal segregation.	P2, P6, P7
Lab 3	Hardy-Weinberg and Microscopes	Modeling allelic distributions in Hardy-Weinberg equilibrium, Genetic drift, Natural Selection using a card-based simulation.	Practicing use of microscopes, micrometers, and calculating magnification	Students manipulate physical models and practice skills regarding scientific instrumentation (microscopes).	P4, P5
Lab 4	Evolution by Genetic Drift and Natural Selection	Modeling allelic proportions under a variety of starting conditions using a computer-based simulation.		Students use a computer-based modeling program to make predictions, view results, and observe statistics.	P1, P2, P4
Lab 5	Phylogenies and the Tree of Life	Building Phylogenies and mapping characters onto a phylogeny		Students use whiteboards and physical models to build and refine phylogenetic trees.	P2, P4
Lab 6	Introduction to Eukaryotes: Evolution and Ecology	Using microscopes to observe and measure cellular structures in eukarya using microscopes	Completing mycorrhizae and Plant growth Experiment (Lab 1)	Students relate observations to biological features and evolution. Students analyze data from a six-week experiment initiated in Lab 1.	P2, P4, P5, P6, P7
Lab 7	Plant Evolution and Ecology	Conducting micro- and macroscopic observations of land plants with regard to structures and life cycles.		Students relate observations to biological features and evolution.	None coded
Lab 8	Fungi Evolution and Ecology	Using dissection microscopes to observe fungal colonies	Completing a write up about their experimental data (out of lab).	Students use observations about fungi to help fill in life-cycle models.	P2, P4, P5, P6, P7
Lab 9	Animal Ecology and Evolution	Observing prepared slides and live specimens with and without compound microscopes		Students identify attributes, diversity, and trade-offs generated by multicellularity by answering questions and mapping traits on a phylogenetic tree.	P4, P6, P7
**BIOL 2**					
Lab 1	Tools of Molecular Biology	Making solutions and serial dilutions, pipetting, using a spectrophotometer		Students use basic math and practice techniques that they will use throughout the course.	P2, P4, P5, P6, P7
Lab 2	Exploring Protein Structure	Examining protein structure and hierarchy using physical and computer-based models.		Students work with 3D physical and computer models of proteins to describe folding, structure, and function	P2
Lab 3	Enzyme Activity	Observing the conversion of substrate to product in the presence of enzyme under various conditions.		Students collect, graph, and analyze data using techniques from Lab 1.	P2, P4, P5, P6, P7
Lab 4	Compound Microscope Techniques	Setting up and using compound microscopes to focus on, illuminate, and measure cells and other objects.	Observing osmosis using algae under hyper and hypo-osmotic conditions	Students use micrometers and different objective lenses to make measurements and observe cellular responses to the environment.	P4
Lab 5	Bacteria as Model Organisms (1)	Practicing serial dilutions, aseptic techniques for bacterial culture, and using spectrophotometry to measure culture density.		Students set up growth plates to observe bacterial cell density and growth. One set of plates is designed to illustrate the genetic basis for biochemical processes.	P4, P5, P6, P7
Lab 6	Bacteria as Model Organisms (2), DNA Sequence Analysis	Observing growth of bacterial variants on different media (from Lab 5)	Using computer-based modeling to explore gene sequences and the consequences of mutation.	Students relate bacterial growth to the presence/absence of substrates and enzymes. Students interact with genetic databases to relate DNA, RNA, and protein sequences.	P2, P4, P5, P6, P7
Lab 7	Viewing Stained Mitotic Cells	Preparing and observing cells undergoing mitosis.		Students use micropipetting and microscopy skills to image dividing plant cells.	P4, P6, P7
Lab 8	Photosynthesis: The Electron Transfer Reaction	Preparing samples for indirect observation of electron transport during photosynthesis.		Students use spectroscopy to gather data on photosynthetic activity under different light conditions.	P4, P6, P7
**BIOL 3**					
Lab 1	Organization of the plant body: Primary growth	Dissecting and labeling of anatomy. Identifying structures on prepared and fresh slides.		Students label images and identify structures related to development and primary growth using compound microscopes.	P6, P7
Lab 2	Organization of the plant body: Secondary growth	Identification of structures on prepared slides.	Planting radish seedlings for experiment on nutrition in Lab 6. Seedling maintenance is ongoing for 5 weeks.	Students use compound microscopes to identify tissues and cell types.	P4
Lab 3	Form and Function in Multicellular Animals	Analyzing videos and prepared slides showing stages of development and illustrating tissue types. Observation of muscle movement in live samples		Students use compound microscopes to identify tissues and cell types. Students record observations about animal movement compared to physical models.	P6, P7
Lab 4	How Do Animals Maintain Homeostasis? Thermoregulation and Respiration	Measuring changes in oxygen consumption in response to changes in temperature.	Completing a write up about their experimental data (out of lab).	Students use a dissolved oxygen meter and a respirometer to measure oxygen consumption of fish and mice.	P2, P4, P5, P6, P7
Lab 5	Animal Nutrition: Digestion and Absorption	Observing feeding and digestive systems in live animals. Dissecting and identifying digestive tissues and structures in fresh and preserved samples.		Students use a dissecting microscope and observe species by eye. Students identify [specialized] structures using illustrations as guides.	P4
Lab 6	Plant Nutrition	Conducting final observations and measurements (length and weight) of radish seedlings grown since Lab 2 with different non-organic nutrition supplements.		Students use pooled results from all BIOL 3 sections to write a report on plant nutrition.	P2, P4, P6, P7
Lab 7	Water Relations in Multicellular Organisms	Viewing prepared slides of leaf and stem plant tissues; measuring transpiration under different conditions using a porometer.	Dissecting and identifying circulatory and gas-exchange tissues in fresh and preserved animal samples.	Students are reminded of principles of osmosis and diffusion at the beginning of the lab session.	P2, P4, P5, P6, P7
Lab 8	Reproduction in Animals and Plants	Bisecting flatworms; identifying of gonads in fresh and preserved specimens of plants (flowers, fruits, and seeds) and animals.		Students dissect and observe specimens by eye and using a dissecting microscope.	P2, P4, P6, P7, P8

**Fig. 2:**
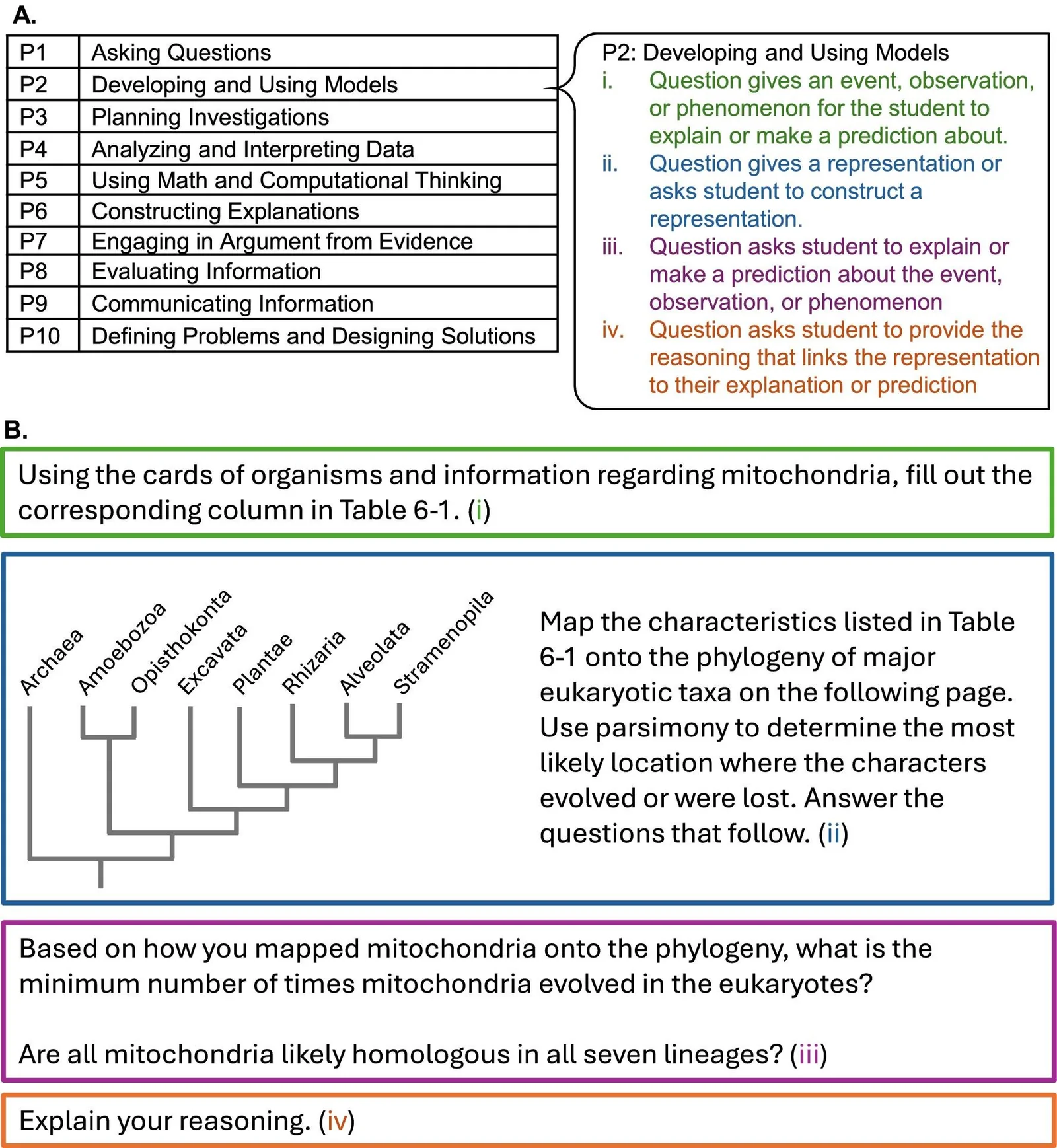
**A.** The science practices put forth by *A Framework for K-12 Science Education* and included in the Next Generation Science standards (left) are defined in the 3D-Learning Assessment Protocol (3D-LAP) by a series of criteria that must be met in order for a student to potentially participate in the practice. At right, the criteria for Science Practice P2, Developing and Using Models (i–iv). **B.** An example of material that addresses all of the criteria for Developing and Using Models. Each of the criteria in part A (i–iv) is noted by a colored box in part B. Students fill out a table of characteristics for each of seven taxa, students map characteristics onto a phylogenetic tree to create a representation of how taxa are related, students are asked to predict how many times mitochondria evolved in eukarya, students are asked to use the logic that they used to create their representation to explain their prediction. Phylogenetic tree image is modified from the BIOL 1 lab manual (Hayden-McNeil, 2019, 2023).

We evaluated the materials for each laboratory session for the potential to elicit one or more practices. Figure 2 lists the ten science practices and focuses on one of them, Developing and Using Models (DUM, P2) and its criteria. Here, we use the example shown in Figure 2 to illustrate our coding process for laboratory material that meets all of the DUM criteria. Briefly, the lab materials (manual, pre-lab, and post-lab) associated with the lab session (BIOL 1, Lab 6) were read all the way through and were assessed as a single unit against the 3D-LAP criteria that define participation in each science practice. In the set of prompts shown in Figure 2, students are prompted to view, measure, and draw organisms and to use “cards of organisms and information regarding mitochondria [to] fill out the corresponding column in Table 6–1,” fulfilling the first DUM criterion, which requires that a context (event, observation, or phenomenon) be provided within which the student will explain or make a prediction (Figure 2a, green text (i), Figure 2b green text box (i)). Students are then prompted to “map the characteristics listed in Table 6–1” onto a phylogenetic tree, fulfilling the second criterion for DUM of asking students to construct a representation (Figure 2a, blue text (ii)). The instructions direct students to use their prior observations and content knowledge in this step (Figure 2b, blue text box (ii)). Next, students are asked to predict “the minimum number of times mitochondria evolved,” and if “mitochondria [are] likely homologous” which fulfill the third criterion of asking students to make predictions about the given observation (Figure 2a, purple text (iii), Figure 2b purple text box (iii)). Finally, students are asked to explain their reasoning. Because students must use their constructed representation to support their predictions “based on how you mapped mitochondria onto the phylogeny” and “all seven lineages,” respectively, this fulfills the final criterion that the “question asks students to provide the reasoning that links the representation to their explanation or prediction” (Figure 2a, orange text (iv)). As all four criteria for DUM are met, this material was coded as having the potential to engage students in this practice. This example also illustrates the importance of coders reading the lab materials for the lab session in its entirety because students encounter the first criterion early in the lab session but then fill out table 6–1 for other cellular attributes and observe, measure, and draw organisms from each taxon, before completing the final three criteria.

Each practice could be coded multiple times in the materials for a single laboratory session and materials that had the potential to elicit more than one practice were coded as including multiple practices. When the materials met all but one criterion for a practice, we coded them as *near misses*, similar to examples identified in other studies (Stowe and Cooper 2017; Uminski et al. 2024; Van Wyk et al. 2025), while those that did not meet more than one criterion were coded as not having the potential to engage students in science practices. When the materials under consideration failed to meet all criteria for any given practice, coders also recorded where and how wording or follow-ups could be revised so that they would elicit a practice.

Although the courses (BIOL 1, 2, 3) comprise both lab and lecture components, in this study, we confined our analysis to the printed materials that were provided to students. These materials included laboratory manuals and pre-and post-lab assignments for each course. When these materials included directions for writing lab reports, we coded those instructions. Lab materials generally included directions for how students should divide work or collaborate on lab activities or experimental protocols. Instructions that were provided outside of these laboratory materials were not considered in our analysis. The BIOL 2 manual includes questions that are posed to students as interstitial “stop-and-think” questions that are not turned in or required for completing the laboratory. These questions were included in the identification of practices in the laboratory courses (a total of 5 of the 45 practices coded in BIOL 2) although there were no explicit instructions to complete these activities, so they could be interpreted by students as *optional* activities.

## Training and calibration

Before coding the full set of laboratory sessions, the research team consisting of three principal investigators (PIs) and two student researchers who had previously taken the introductory laboratory series, established a shared understanding of the coding protocol (3D-LAP) and what constituted evidence that the criteria for each science practice had been met. Calibration occurred over three rounds. In Round 1, each coder independently coded the same laboratory session based on their interpretation of the protocol. The full research team then met to compare code assignments and discuss the rationale for determining whether the criteria for each science practice had or had not been met. In Round 2, each coder independently coded two laboratory sessions before meeting in small groups of two to three coders to compare code assignments. These discussions were followed by a whole-group meeting to further examine coding decisions and ensure consistent interpretation of the protocol. A third calibration round followed the same process as Round 2, allowing coders to gain additional experience and confidence in applying the protocol before beginning full-scale coding. The coding protocol remained unchanged throughout calibration. Instead, multiple rounds served to improve consistency in coders' interpretation and application of the protocol.

## Assessment of coding reliability

Inter-rater reliability was calculated prior to the whole-group discussion in the third calibration round. The overall % agreement among the five coders across the independently coded laboratory sessions was 87% and Fleiss' kappa was 0.62, indicating substantial agreement (Bowen et al. 2025; Landis and Koch 1977). Based on these results, the research team proceeded to code the full dataset.

## Coding of the full dataset

Following calibration, coders began coding the full set of laboratory sessions. Individual coders independently analyzed materials from two to three laboratory sessions before meeting in small groups of two to three coders to compare, discuss, and reconcile coding differences. The results of each small-group consensus meeting were documented separately and presented during weekly or biweekly meetings of the full research team. These meetings provided an additional opportunity to review coding decisions for consistency and alignment with the protocol. The coding process was iterative, with discussions throughout the project informing consistent application of the protocol, and final code assignments were established through whole-group consensus. Coding of the three introductory laboratory series was completed over approximately 24 months.

## Results

**Fig. 3:**
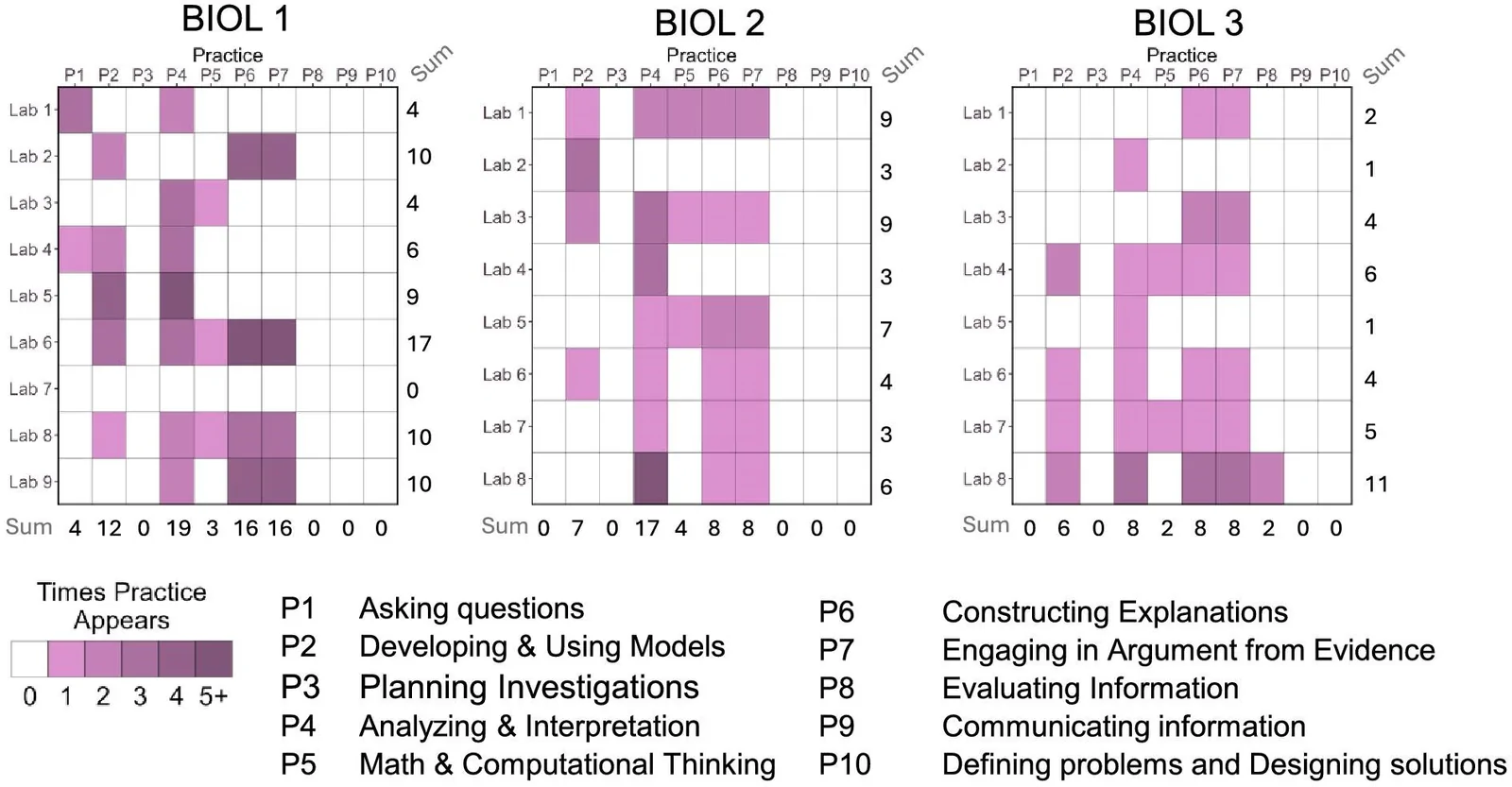
Presence and absence of science practices in a three-course series of introductory biology lab sessions. Lab manuals were coded for activities that have the potential to elicit science practices as shown in the heatmaps (BIOL 1, left; BIOL 2, center; BIOL 3, right). X-axes show the 10 science practices, P1-P10 (see key, at right); Y-axes indicate the Lab session that was evaluated. Darker shading indicate more instances of a given practice being coded as present (see key, at right). Activities that did not meet all of the criteria required to potentially elicit a science practice were not counted and white (unshaded) squares indicate that a practice was not present in a given lab session. Numbers on the far right of each heat map show the sum of all of the coded items for each lab session. Numbers at the bottom of each heat map show the sum for each practice in each course.

We examined the laboratory materials (manuals, pre-lab and post-lab assignments) for 25 laboratory sessions across the three introductory biology courses for evidence of potential to engage students in the science practices. Nearly all (24/25) laboratory sessions showed evidence of the potential to engage students in at least one practice, and 20/25 included the potential to engage students in more than one practice (Figure 3, Table 2). As noted in the methods, we determined that a practice could be elicited when all the criteria for that practice were met.

**Table 2:** Distribution of items (comprised of questions, sets of questions, procedures and/or follow ups found in lab manuals) that were coded as having the potential to elicit science practices in BIOL 1, BIOL 2, and BIOL 3 in-lab versus out-of-lab.

	**BIOL 1**	**BIOL 2**	**BIOL 3**
In lab	52	30	14
Out of Lab	19	15	22
Total	71	45	36

### Presence and absence of science practices in introductory biology labs

Across all three courses we found that there was strong emphasis on Analyzing and Interpreting Data (80% of sessions), Constructing Explanations (64% of sessions), and Engaging in Argument from Evidence (64% of sessions). This pattern was emphasized in the first course of the series, BIOL 1. Over all the lab sessions, three practices–Developing and Using Models, Using Math/Computational Thinking, and Asking Questions–were identified at lower frequencies (52%, 32%, and 8%, respectively). Three practices–Planning Investigations, Communicating Information, and Defining Problems/Designing Solutions–were not identified in any of the biology laboratory sessions. In total, we identified 152 opportunities to engage in science practices across the introductory biology lab series. BIOL 1 had the largest proportion of opportunities to engage students in science practices (71 (46% of the total)), while BIOL 2 included fewer (45 (29% of the total)), and BIOL 3 the fewest (36, (24% of the total)). Interestingly, while BIOL 3 provided the fewest opportunities for students to engage in science practices (50% fewer than BIOL 1), this course provided the opportunity for students to engage in the widest variety of practices, being the only course in the series that includes Evaluating Information.

### Distribution of science practices in and out of the laboratory classroom

**Fig. 4:**
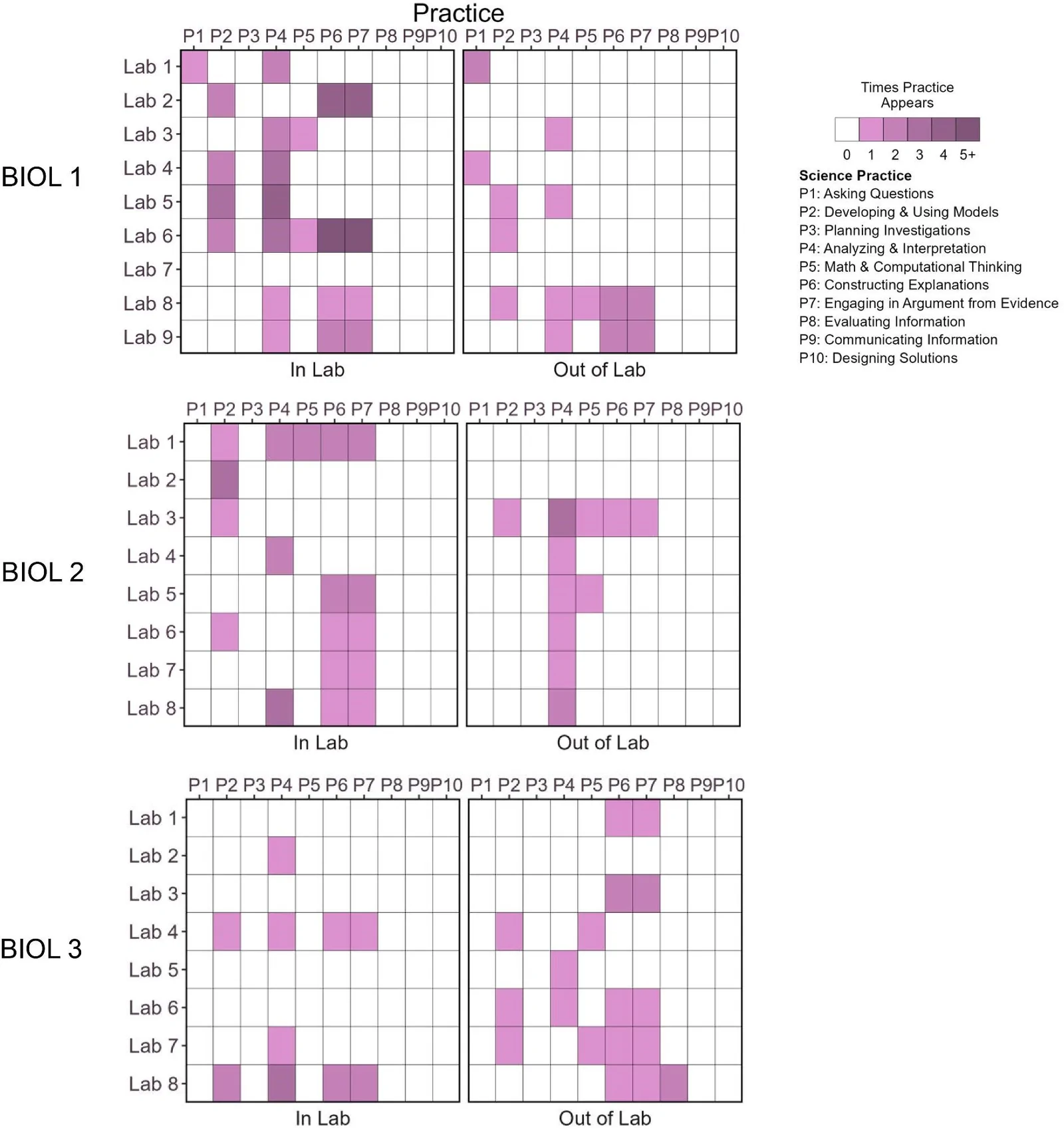
Distribution of science practices between in-class activities and out-of-class assignments. Activities that were coded as having the potential to elicit science practices are shown in the heatmaps (BIOL 1, top; BIOL 2, middle; BIOL 3, bottom). Coded instances are shown using the same scale as in Figure 3.

We were interested in whether practices were distributed evenly between the in-class laboratory sessions and out of class assignments (including worksheets that might have been initiated in the lab but could be completed afterwards). To determine this, we divided our data and graphed it according to whether the coded practices occurred during lab-classroom times, or if students would have encountered the practice in a pre- or post- lab worksheet (Figure 4, Table 2).

In BIOL 1, 52 (73%) of the 71 opportunities to engage with a science practice were identified as part of the laboratory materials completed during the lab period. The remaining 19 (27%) opportunities were identified in out-of-lab activities. In BIOL 2, we found a similar pattern, with 30 (67%) of 45 opportunities coded for the in-lab activities and 15 (33%) as out-of-lab activities. Interestingly, the pattern was reversed in BIOL 3, where we identified 14 (39%) in-lab of the 36 opportunities for participation in science practices in in-lab activities compared with 22 (61%) out-of-lab (Table 2).

In both BIOL 1 and 3, there were more opportunities to engage students in science practices out of lab towards the end of the course, compared with the beginning of the course. Additionally, we found that many out-of-lab opportunities were coded as Constructing Explanations and Engaging in Argument from Evidence. These were generally questions or groups of questions that asked students to draw connections between content knowledge and their observations from the in-lab session. Developing and Using Models and Analyzing and Interpreting Data were also more highly represented in out-of-class materials in the later sessions of the courses. In these sessions, students spend the in-lab time collecting a full data set, but do not have scheduled time to analyze or interpret that data during the lab period. In BIOL 2 we also identified consistent instances of Analyzing and Interpreting Data (for example analyzing conversion rates in enzymatic reactions, Labs 4 and 8) in the out-of-lab activities, but we did not find an overall increase in out-of-lab science practices.

### “Near misses” in BIOL 1, 2, and 3

**Fig. 5:**
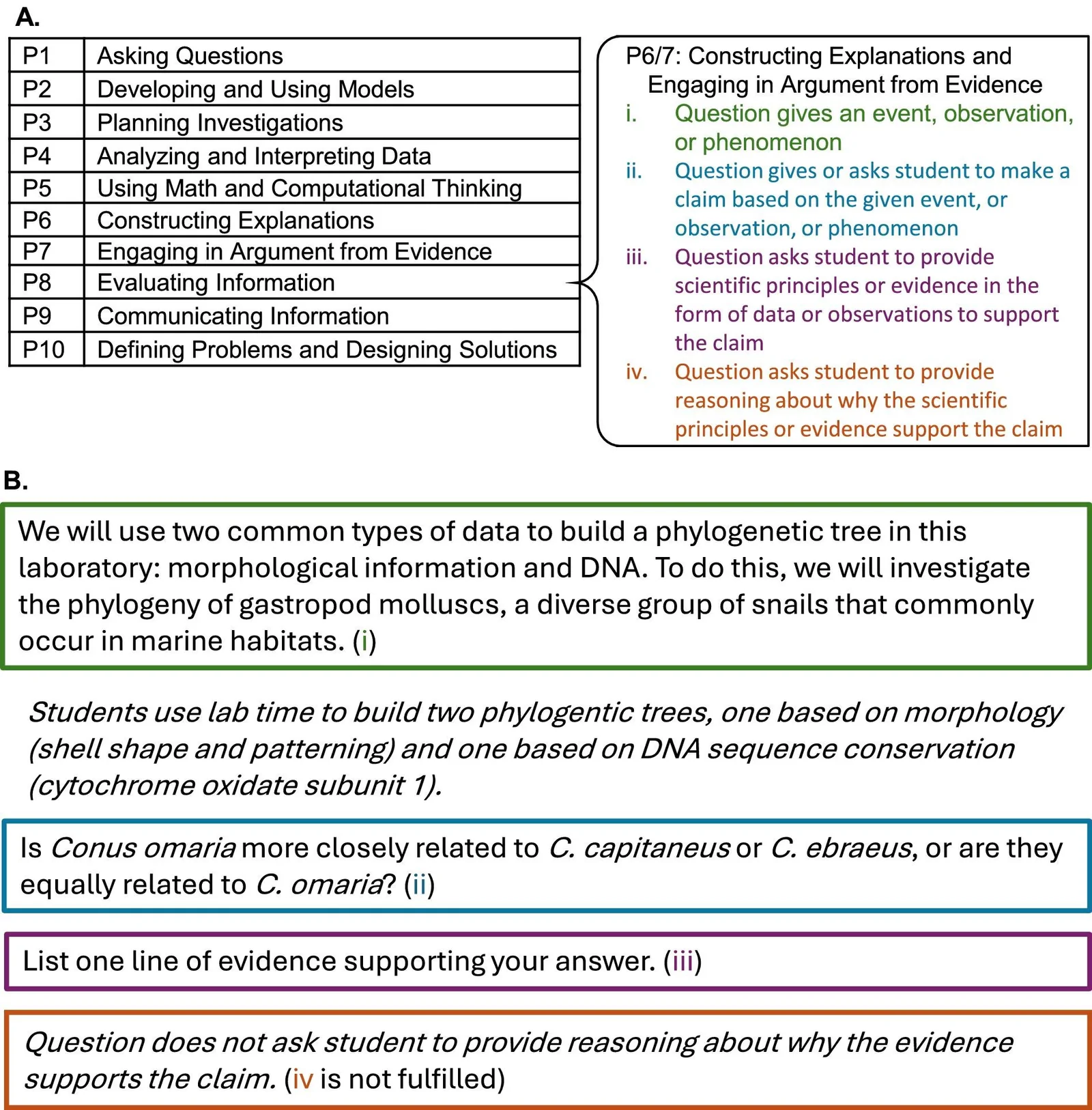
**A.** The science practices put forth by *A Framework for K-12 Science Education* and included in the Next Generation Science standards (left), as in Figure 2. At right, the criteria for Science Practices P6/7, Constructing Explanations and Engaging in Argument from Evidence (i–iv). P6 and P7 are coded with the same criteria (Laverty et al. 2016, Carmel et al. 2019). **B.** An example of material that addresses all but one of the criteria required to elicit the practices. Each of the criteria in part A (i–iv) is noted by a colored box in part B. The manual describes a set of molluscs that are related. Students use morphology and DNA sequence analysis to build two phylogenetic trees that show the relationships between the species, students are asked to make a claim about which species are most closely related, and they are asked for a piece of supporting evidence. Students are *not* asked to provide reasoning about why the evidence supports their claim.

**Fig. 6:**
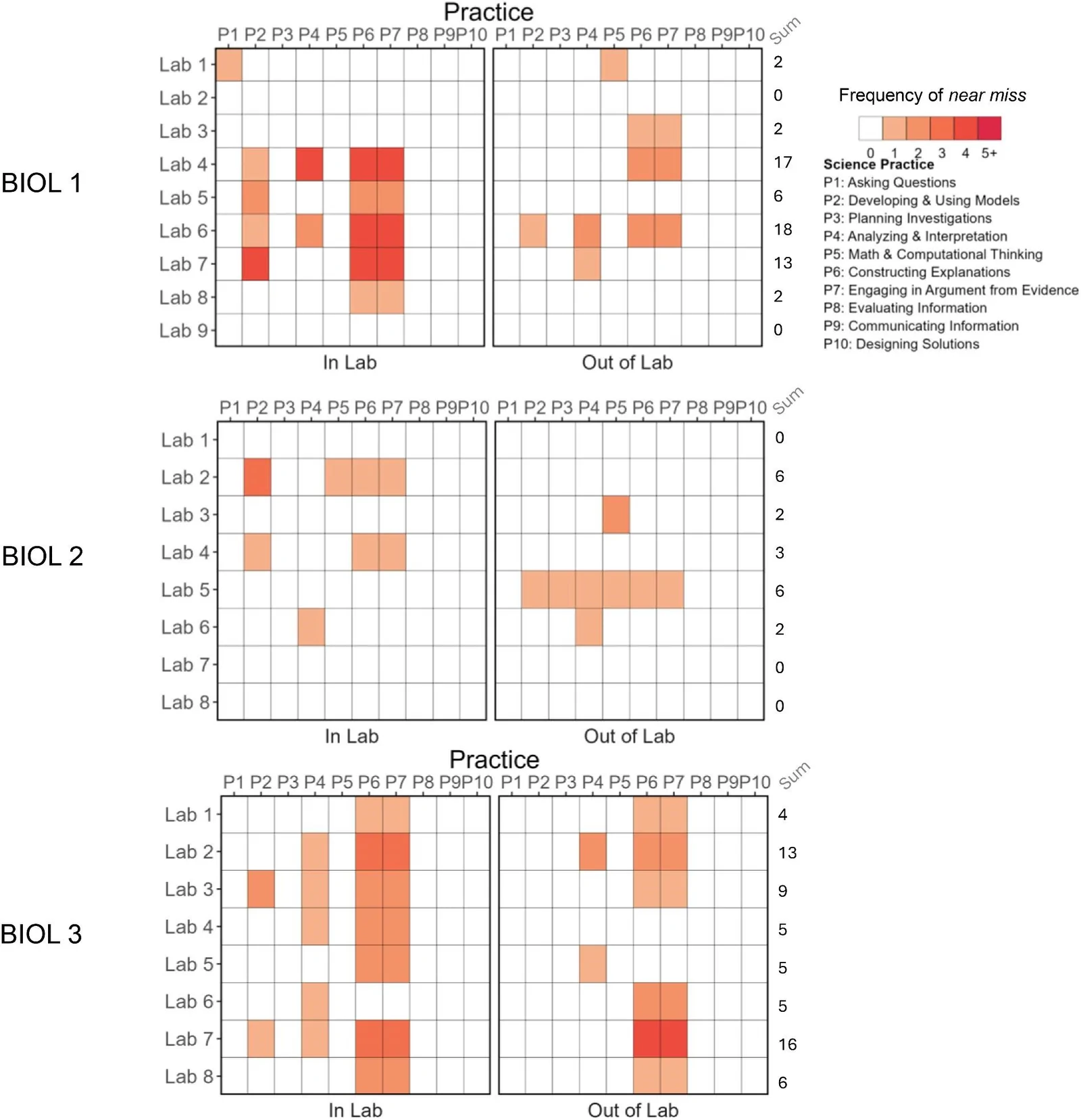
The presence and absence of “*near misses*” in and out of lab, by course. Coded instances that meet *all but one* criterion for having the potential to elicit science practices are shown. “Near misses” were categorized by whether they were included as part of the in-lab session, or as out-of-lab homework (prelab, post-lab, etc.). Numbers on the far right of each heat map show the sum of all of the coded items for each lab session.

**Fig. 7:**
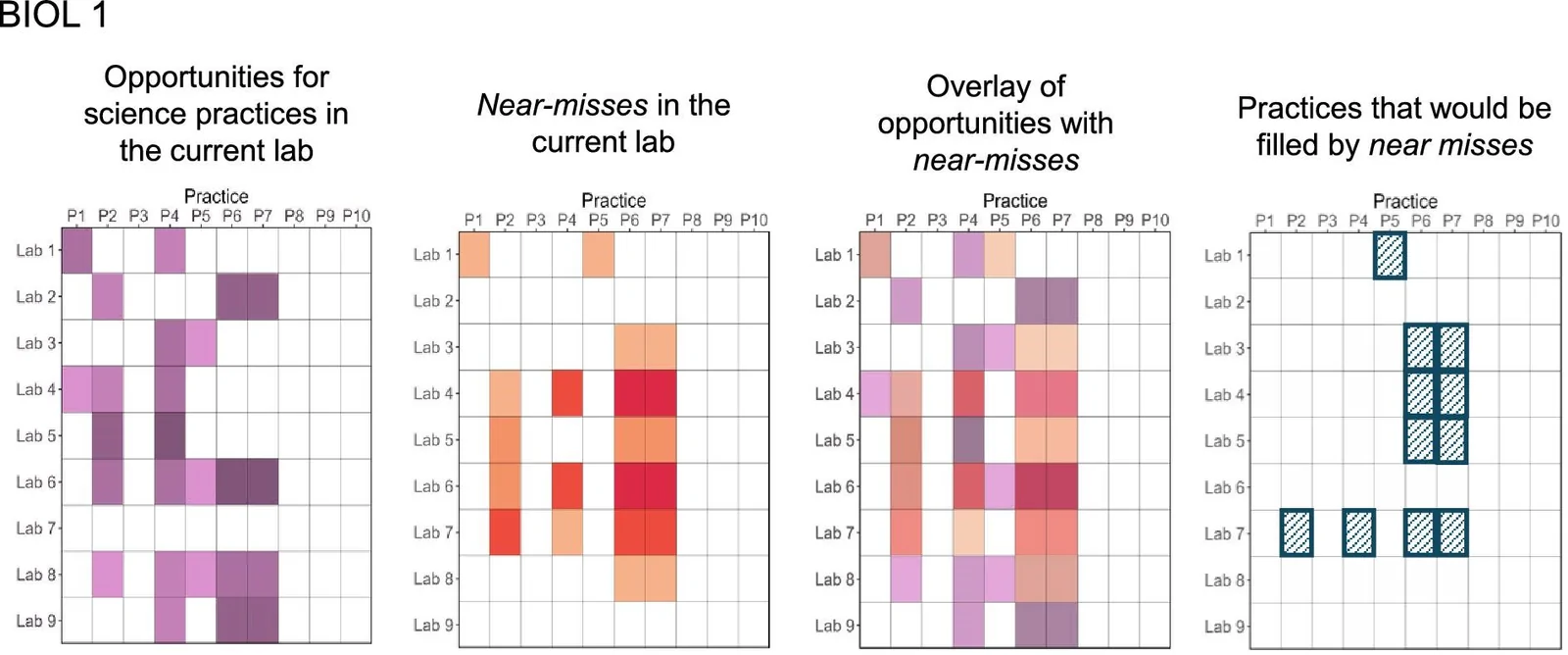
Overlay of the presence of science practices in the current lab manual with the *near misses* in the same manual. From left, the presence and absence of opportunities for students to engage in science practices in the current lab materials; the *near misses* coded in the materials; the two heat-maps overlayed to show how the curriculum could be “filled in” by modifying near misses to potential science practices (merged). Far right, activities that could introduce a practice where there is currently *none* are shown as hatched squares, right.

In coding the laboratory materials (manuals and pre- and post-lab assignments), we assessed lab materials to determine if they met the criteria set forth by the 3D-LAP. We noted instances where most, but not all criteria were met and called these instances *near misses* (Figure 5, Table 3). *Near misses* lacked only one criterion, and usually they were missing the final reasoning/consequence component. Figure 5 shows an example of a *near miss* for the practices Constructing Explanations and Engaging in Argument from Evidence. Figure 6 shows how *near misses* are arrayed across the three courses, with attention to whether they would be performed during the lab (in-lab) or before or after lab (out-of-lab). BIOL 1 includes 59 total *near misses* (44 in lab, 15 out-of-lab), and BIOL 2 and 3 includes 18 (10 in lab, 8 out-of-lab), and 60 (39 in lab, 21 out-of-lab), respectively. *Near misses* “fill in gaps” in several lab sessions, which suggests that if the items that did not fulfill all the criteria prescribed by the 3D-LAP were modified such that they did fulfill all the criteria, some labs would then include instances of science practices that were not previously identified (Figure 7). This is particularly evident in the case of BIOL 1, Lab 7, which does not currently show potential to elicit any science practices, but has *near miss* opportunities for four different science practices. Modifying these activities or questions so that these *near misses* are turned into actual opportunities for engagement in science practices would mean Lab 7 now has the potential to engage students in four science practices. Similarly, modifying *near misses* in Labs 3, 4, and 5 in BIOL 1 would add opportunities for students to participate in Constructing Explanations and Engaging in Argument from Evidence (Figure 7). Modifying *near misses* so that they could elicit science practices would significantly alter the landscape of BIOL 1 so that students would have 59 (83%) more opportunities to engage in science practices in the BIOL 1 lab series. Modifying *near misses* would also increase the number of times students encounter opportunities to engage in practices in BIOL 2 (18, or 40% more) and 3 (60, or 167% more), but we found only a few instances where science practices that are not currently coded for at all could be introduced by modifying the existing curriculum (BIOL 2, Lab 3; BIOL 3, Labs 2 and 5), Supplementary Fig. 1).

**Table 3:** Distribution of items (comprised of questions, sets of questions, procedures and/or follow ups found in lab manuals) that were coded as *near misses* that do not meet one of the criteria required to elicit science practices. Items are shown by what course they were from (BIOL 1, 2, or 3) and if they were in-lab versus out-of-lab

	**BIOL 1**	**BIOL 2**	**BIOL 3**
In lab	44	10	39
Out of Lab	15	8	21
Total	59	18	60

## Discussion

In this analysis of an introductory biology laboratory series, we found evidence for the science practices laid out in the NGSS and *The Framework* in nearly all laboratory sessions. Practices were not evenly distributed across the courses or lab sessions, and not all of the practices were represented in this course series. The three linked courses (BIOL 1—Introduction to Evolution, Ecology, and Biodiversity, BIOL 2—Introduction to Cellular and Molecular Biology, BIOL 3—Introduction to Organismal Biology) have distinct content goals, but the series should provide a general introduction to the biology content and competencies described in *Vision and Change*. It is therefore heartening that all three courses include science practices, which require students to combine content knowledge and skills, in their laboratory materials.

Based on our analysis, some elements of doing science are absent or underrepresented in this introductory biology curriculum. For example, students are not regularly expected to engage in Asking Questions or Planning Investigations, though these are at the heart of scientific inquiry (Figures 3 and 6). In addition, Evaluating Information and Communicating Information are rarely if ever included, largely because the criteria (“gives an excerpt from a conversation, article, student solution, or video” and “communicate their findings to an audience beyond the instructor in a way that could be understood by the general public,” respectively) are not commonly included in this curriculum. However, these skills are arguably more important now than ever as students need to be able to find and assess sources of trustworthy information and describe the importance of scientific endeavors to outside audiences. Indeed, Barnes et al., found that students lack confidence in speaking about complex science topics and suggested that this could be ameliorated with more concrete practice in the lab classroom (Bowen et al. 2025). Several groups have identified ways in which “traditional” curricula in classrooms and laboratories could benefit from intentional redesign or modification using the 3D-LAP criteria, which can significantly increase opportunities for students to engage with science practices in general, and with low-frequency practices in particular (Uminski and Couch 2024; McFall-Boegeman et al. 2025; Bolland et al. 2025; Stephenson et al. 2026). While we are not suggesting that students should engage in all science practices in each laboratory session, we believe that the full introductory series of courses should provide students with opportunities to do science authentically, which demands engagement in all science practices.

Our analysis of *near misses* also suggests ways that more of these practices could be included. We coded activities as *near misses* when all but one of the 3D-LAP practices criteria were met. As in other studies, we often found that the “missing” criterion required students to address their reasoning or otherwise justify their answer (Stowe and Cooper 2017; Uminski and Couch 2024). This means that students could fully (and correctly) answer a question without reflecting on their reasoning and “fully engaging in a scientific practice” (Uminski and Couch 2024). We found that nearly all these instances could be revised relatively easily and without changing the overall purview of the lab. Supplementary Table 1 shows examples of how changes to the wording of instructions or questions could allow them to provide opportunities for students to engage with one or more science practices. However, it can be challenging to meet some of the criteria for the science practices required by the 3D-LAP without intentional [re]design of a curriculum. For example, one criterion for Communicating Information is for students to “communicate their findings to an audience beyond the instructor in a way that could be understood by the general public.” This criterion will require planning so that students have a time and place to present in a way that is dissimilar to the style of their lab materials. Likewise, Asking Questions and Planning Investigations are somewhat impeded because students are provided with the topic or inquiry as well as the complete protocol for lab activities or experiments. Revisions could be made that would engage students in taking an active role in choosing from (for example) a palette of questions or possible technical approaches. This would also require planning and buy-in from both instructors and support staff, who may be reticent to relinquish control, especially in a laboratory environment where safety is critical (Shortlidge et al. 2017; Heim and Holt 2019; Govindan et al. 2020). However, if simple language changes (for example, “use the information on your diagram to explain your reasoning”) could fulfill a final criterion, students could have more opportunities to engage in science practices without major overhaul to a curriculum.

In this curriculum, we noted a drop in the number of science practices as students progressed through the courses (71à45à36 science practices in BIOL 1, BIOL 2, and BIOL 3, respectively). Based on the activities in the labs (Table 1), students are not necessarily tackling more technical practices as they progress in the courses. In some cases (for example, BIOL 1, lab 9 and BIOL 2, lab 5), there is relatively little new technical skill involved, but students use lab time to collect observations in a variety of ways to generate data or representations. These practical applications could contribute to Analyzing and Interpreting Data, Constructing Explanations, Engaging in Argument from Evidence, and Developing and Using Models, which are practices that we identified relatively frequently. In other lab sessions (for example, BIOL 1, lab 6; BIOL 2, lab 4; BIOL 3, lab 4), students engage with new or relatively complicated techniques. It does not appear that the introduction of new techniques correlates with the number of items coded (Figure 3). However, they reiterate practical knowledge (for example, using microscopes, looking at DNA and protein sequences, and graphing data), and content knowledge (for example, chromosome segregation, requirements for plant growth, cellular structures and organismal body plans), which is not a science practice, per se, but is essential for participation in science practices (National Research Council 2012; National Research Council 2013; Laverty et al. 2016). Future work could consider if the topics covered in the later laboratory sessions need more significant modification to fully engage students in the process of science as it relates to them, or if research-oriented modules could be introduced as ways to foster engagement with different science practices (Lee et al. 2019; Dahlberg et al. 2019; Buchanan et al. 2022).

We noticed that opportunities for students to engage with science practices during the lab session itself (“in-lab”) also dropped from the beginning of the curriculum to the end. In BIOL 1, BIOL 2 and BIOL 3, 73%, 60%, and 38%, respectively, of the instances of science practices that we coded occurred in materials that students would be most likely to encounter in the lab settings. The remaining practices would be performed as pre- or post-lab materials. In BIOL 3, for example, students are often asked to examine many specimens to draw conclusions about similarities and differences between them (Table 1). While this does not require novel skills, per se, it does require attention to detail and time. We therefore see that there are fewer instances of the coded practices, and that they tend to be in items that are completed outside of the lab itself (Figure 4). In addition, over the entire curriculum, the percentage of *near misses* (where a slight modification would make it more likely that a student would participate in a science practice) that were part of the in-lab materials decreased from 75% in BIOL 1 to 56% in BIOL 2 and 65% in BIOL 3.

As students progress through the course series, they are expected to prioritize lab time for practicing technical skills. As a consequence, they are expected to take time to do science practices outside of lab, while competing with class schedules and extracurricular responsibilities–including jobs that can make it very difficult for lab partners to schedule time to work together (National Academies of Sciences, Engineering, and Medicine 2016; Hsu et al. 2022; Navarro et al. 2025). This is of concern to us because science practices are not just skills (what one can do) but integration of content knowledge and skills and scientists often do this type of integration collaboratively. Although it is implicit in *The Framework* (National Research Council 2012), successful engagement in science and science practices benefits from students polling ideas of relevant concepts, exchanging reasonings and explanations, and reflecting on their own thinking (Corwin et al. 2018). Much of this is best supported by the collaborative nature of laboratory settings, where students are learning with peers, near-peer instructors and instructors–interactions that often catalyze deep thinking and reflection (Freeman et al. 2014; Snyder et al. 2015; Stephenson et al. 2019; Theobald et al. 2020; Arvelo-Márquez et al. 2021; Clements et al. 2022).

Finally, student accountability can wane when there is not clear value placed on an activity (Seery et al. 2024; Huseeb et al. 2026). In this study, activities or questions that appeared in the lab manual but were not a requirement for completing the lab session were coded for practices. However, student coders reported that completion of these activities might have caveats. For example, five of the items in BIOL 2 that were coded as Constructing Explanations and Engaging in Argument from Evidence required that students “be ready to explain” or give an explanation, without requiring students to either actively seek feedback or write answers down, respectively. Student coders noted, and we have since observed (data not shown), that when students have opportunities to participate in practices, but are not explicitly prompted to or held accountable for doing so, they often will not. We suggest that when manuals include opportunities to participate in a practice during the lab session, those opportunities should be facilitated, either by requiring students to write or turn in their answer, or by asking them to discuss their responses with an instructor.

## Implications for teaching and learning and researchers

Our findings from assessing the introductory biology lab curriculum prompt us to reflect on two foundational questions: *What do we want our students to learn?* and *What does this mean for instructional planning?* Underlying these questions is the need for intentionality in curriculum design. Specifically, what we value in science learning and teaching (that is, our pedagogical priorities) should shape how curricula are structured. If we agree that engaging in science practices is an important learning outcome, then our study suggests that these practices must be intentionally incorporated into curriculum, rather than assumed to be emergent.

In our study, we found that many *near misses* only lacked a clear requirement for students to describe their reasoning or concretely draw a connection between two answers or concepts. By assessing whether lab manual items have the potential to meet the criteria that instructors are aiming at, instructors may identify ambiguous or missing prompts and revise them to more directly induce students to participate in science practices. Our findings suggest that these relatively small revisions are low-hanging fruits that are bite-sized and effort-appropriate for individual instructors which could substantially increase the opportunities for engagement in science practices in their own classes. While our work underscores how *near misses* represent accessible and compelling starting points for instructors to make small changes to lab curricula to engage students in science practices, it also highlights the fact that there are some science practices that are not addressed by the curriculum even when *near misses* are taken into consideration. In such cases, more intentional and broader revisions of the curriculum are needed to incorporate these practices.

To support opportunities for students to engage with science practices, we suggest that the 3D-LAP is a useful and practical tool in curriculum design and redesign that can be applied at the individual, small group, and departmental levels. At the individual level, the 3D-LAP criteria provide concrete, step-by-step guidance for instructors to plan learning activities as well as to assess whether an existing learning activity will enable students to engage with a science practice (Laverty et al. 2016; Carmel et al. 2019). This is a different approach from the Laboratory Course Assessment Survey (LCAS) and Colorado Learning Attitudes About Science Survey (CLASS), which assess the experiences of students in biology laboratory courses, but focus on the perceptions of scientific inquiry as opposed to the laboratory materials themselves (Semsar et al. 2011; Corwin et al. 2015). Because each criterion can, in principle, be met independently of the others, instructors can build or revise given prompts around criteria, one at a time, toward a targeted science practice.

While individual instructors can use the 3D-LAP to better design their courses for greater student engagement in science practices, we believe this would be an arduous route. Our experience using the 3D-LAP throughout this study suggests that the protocol is most effective when used collaboratively. We recommend that small teams of instructors, especially those who teach the same course and share curricular materials, work together to interpret the protocol and use it to develop or revise their curricula. We also suggest that disciplinary teams include education researchers who can provide support as they undertake this process. In this way, collaborative effort can reduce the burden on individual instructors while allowing more refined interpretations of the criteria and the prompts from multiple perspectives. More broadly, we suggest that this specific framework can help facilitate conversations among departmental stakeholders about program assessment and responsible, cohesive curriculum. If the programs value student engagement in science practices, then achieving meaningful program-level learning outcomes likely requires a coordinated effort across courses rather than isolated changes within a single class.

The parallels between the three-dimensional learning model and *Vision and Change* (Figure 1) mean that students in undergraduate biology courses that are designed with either framework in mind will likely achieve some or many of the goals of the other. A strength of the 3D-LAP is that it provides a set of criteria that can guide instructors toward meeting a particular concept or practice. Tools designed around *Vision and Change’s* Core Concepts and Core Competencies allow biology teaching programs to assess the extent to which their students have learned and are comfortable working with the Core Concepts (Couch et al. 2017; Summers et al. 2018; Couch et al. 2019; Semsar et al. 2019). The BioSkills Guide and curriculum mapping tool (Clemmons et al. 2019; Clemmons et al. 2020) help define a set of program and course level learning outcomes for the *Vision and Change* Core Competencies. The 3D-LAP criteria for science practices could be helpful in designing materials to include science practices that support those outcomes and to evaluate activities that help students build toward course and program level learning outcomes.

Our work examined science practices in laboratory settings, but participation in science practices is not limited to laboratory environments. However, a survey of biology assessments showed that instructors often do not focus on science practices in lecture/classroom assessments (Uminski et al. 2024), which suggests that lab sections could be an important setting for students to engage in science practices. However, if lecture and lab courses are run independently (for example in BIOL, 1, BIOL 2, BIOL 3, whose lecture and labs are usually taught by different instructors), the collaborative planning and design described above is even more important to ensure alignment and inclusion of science practices that might not be offered or reiterated in lectures.

### Ongoing work

While our current study focuses on the potentiality of student’s engagement in science practices by identifying opportunities for engagement within the laboratory curriculum, we hypothesize that not all opportunities are taken and actualized in students’ laboratory participation. As such, we have begun examining the alignment between the opportunities for science practices embedded in the curriculum and students’ actual engagement in those practices during laboratory activities. Because laboratory settings are often dynamic learning environments rich with student-student and student-instructor interactions, these interactions may help elicit and facilitate student engagement in science practices. While our study is underway, much additional work is needed to better understand how curricular opportunities for science practices translate into student engagement.

## Conclusion and future directions

We found that a traditional laboratory curriculum has the potential to elicit science practices and that minor modifications could increase both the frequency and coverage of the science practices without a major overhaul of the curriculum. Our analysis of three courses in an introductory biology series reveals that many, but not all science practices are represented, and our identification and evaluation of *near misses* point to opportunities to engage students in a greater range of science practices without overhauling the curriculum. This is important given realities of limited time resources for curriculum reform. Future work could focus on assessing a non-traditional lab curriculum (inquiry-based, CURE, etc.) to see if they provide more opportunities for students to engage in science practices (Brownell and Tanner 2012; Govindan et al. 2020). However, our study shows that smaller reforms that adjust language can integrate science practices in the curriculum, even if there are limited resources for more substantial reforms, such as moving away from traditional labs to non-traditional labs. The science practices criteria described in the 3D-LAP also provide clear and concrete guidance for assessing student development of core competencies described in *Vision and Change*. Thus, designing curricula with the 3D-LAP criteria in mind can help prepare biology students for graduation and all students for participation in the post-graduation world.

This study was limited to analyzing written lab artifacts (manuals, worksheets, assignments), and in the future we will focus on determining whether the potential to engage in a practice translates to participation in a practice. Future work could also examine how, if at all, instructor pedagogy and presence of peer instructors bring about more or fewer opportunities for students to engage in science practices. Nonetheless, providing the opportunity for students to engage in science practices is a crucial first step towards having them participate as fully as possible in the scientific enterprise.

## Supplementary Material

obag049_Supplemental_Files

## Data Availability

Data sets can be made available upon request.

## Footnotes

1 The practices Constructing Explanations and Engaging in Argument from Evidence have the same criteria in the 3D-LAP and are therefore coded simultaneously (Carmel et al. 2019).
