# Supplementary material for: Examining Science Practices in an Introductory Biology Laboratory Curriculum
Source: Integr Org Biol. 2026 Aug 18;8(1):obag049. doi: 10.1093/iob/obag049 (PMC13548873; doi:10.1093/iob/obag049)
Supplement: Supplementary file 1 [file obag049_supplemental_files.zip › Figure S1.pdf]

Supplementary Figure 1

BIOL 2

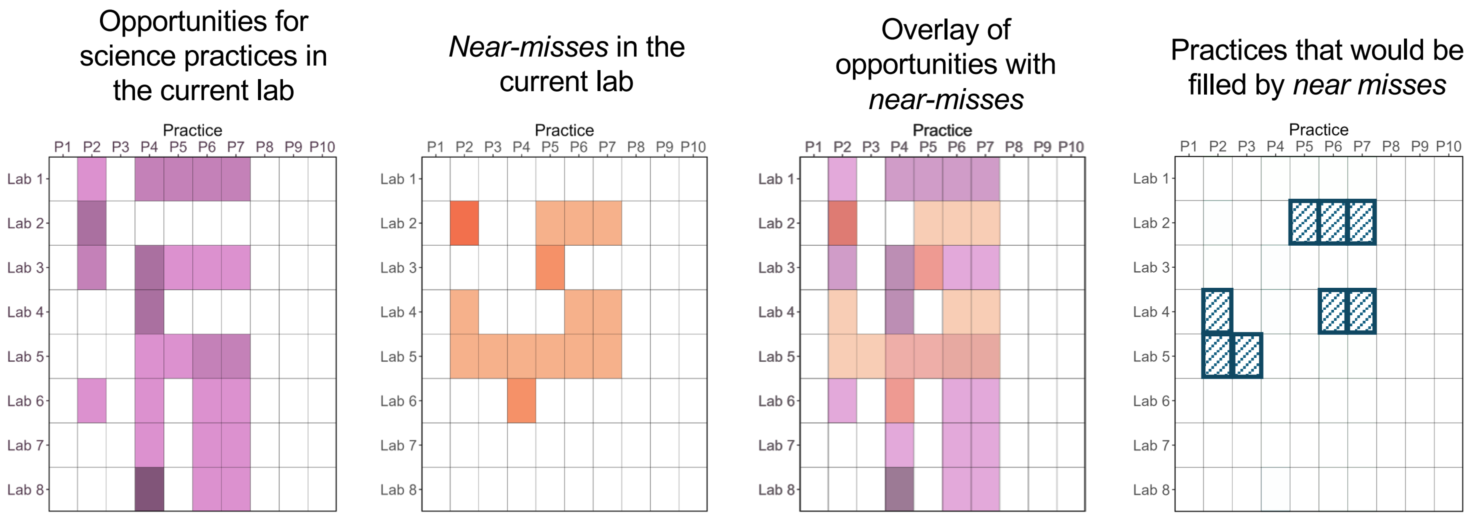

BIOL 3

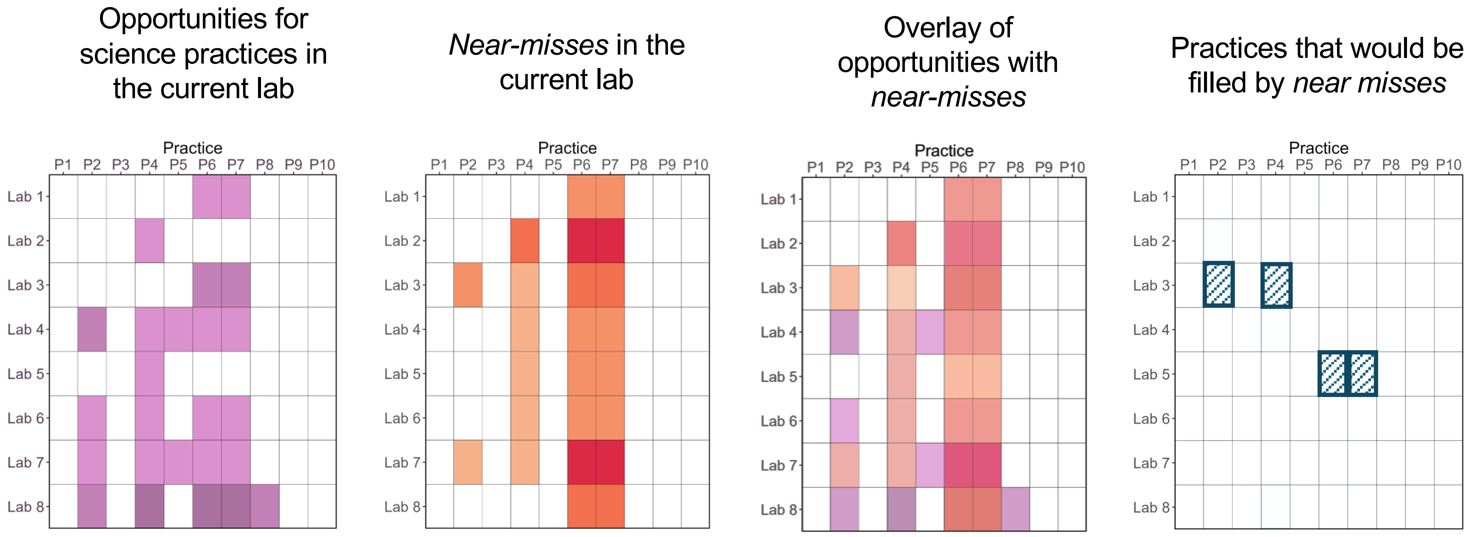

Supp. Fig. 1 Overlay of the presence of science practices in the current lab materials with the *near misses* in the same materials, for BIOL 2 and BIOL 3. Heat maps and hatched patterns as in Figure 7.
