# Supplementary material for: Examining Science Practices in an Introductory Biology Laboratory Curriculum
Source: Integr Org Biol. 2026 Aug 18;8(1):obag049. doi: 10.1093/iob/obag049 (PMC13548873; doi:10.1093/iob/obag049)
Supplement: Supplementary file 1 [file obag049_supplemental_files.zip › Supplementary Table 1.pdf]

Supplementary Table 1. Examples of activities that were coded as near misses, with suggestions for how they could be modified to achieve all of the 3D-LAP criteria for stated practices. These examples each meet all but the final criterion for a practice, which often asks students to describe reasoning or other justification.

Context	Question	Practice *near miss*	Suggestion
**BIOL 1**			
Students use a simulation to model the consequences of allele frequency (HbA vs HbS, in the human hemoglobin gene) and phenotype on genetic drift and natural selection (based on sickle cell vs malaria infections).	What will happen to the proportions of each allele if the symptoms of sickle cell anemia are cured with a drug this year? In other words, people with the HbS allele still have sickled red blood cells, but the adverse effects of the condition (death) can be eliminated with the drug. Adjust the "anemia death rate" but use the actual malaria death rate for your country.	*Analysis and interpretation of data (P4).*<br><br>Students are not asked to "interpret the results or assess the validity of the conclusions"	After determining the result of the simulation (analysis), students could be asked to interpret what the driving factor(s) are (interpretation).
**BIOL 1**			
Students are asked to observe an organism (Red algae) under a microscope and draw an image of what they see, recording the organism name, total magnification, and including a scale bar.	Though closely related to green algae, red algae are very different in color.<br><br>Speculate on why that might be. How might this difference influence the depth of water at which these algae can live?	*Constructing explanations and Engaging in Argument (P6, P7)*<br>Students are not asked to "provide reasoning about why the scientific principles or evidence support the claim."	Students could be asked to explain their reasoning based on their knowledge of cellular compartmentalization and chlorophylls.
	Are red algae photoautotrophs or chemoorganoheterotrophs? Explain your reasoning.	*Developing and using Models (P2)*<br>Students are not asked to "provide the reasoning that links the representation to their explanation or prediction."	Students could be asked to explain their reasoning by using the drawings of the cellular shapes and compartments that they drew.
**BIOL 2**			
Students complete a exercise that requires them to fold a "polypeptide" model according to principles of chemistry/biochemistry.	How many different proteins (15 amino acids long) could you make given an unlimited number of each of the 20 amino acids?<br><br>The average protein is about 300 amino acids long. How many different possible proteins (300 amino acids in length) could exist?<br><br>There are many fewer (orders and orders of magnitude fewer) actual proteins than possible ones. Why do you think there are fewer actual proteins than possible ones?	*Using math and computational thinking (P5)*<br><br>Students are not asked to "give a consequence or an interpretation (not a restatement) … using reasoning…."	Students could be asked to find the consequence of their calculation (there are fewer actual proteins compared to the calculated possible number), instead of being given the consequence as part of the third question.
**BIOL 3**			
Students are asked to identify the confounding variables in a quarter-long plant nutrition experiment that they have analyzed.	Suggest two ways in which we could redesign this class experiment to reduce the influence of the confounding variables.	*Planning investigations (P3)*<br>Students are not asked to "justify how their description, design, or observations can be used to answer the question or test the claim or hypothesis."	Students are given a hypothesis and problem. They could be asked to justify their redesign idea.
